# Development of in situ real-time sensor for molecular contamination

**DOI:** 10.1038/s41598-025-09312-4

**Published:** 2025-07-11

**Authors:** Lasserre Alexis, Simon Léo, Eck Julien, Rossignol Jérôme, Dupont Céline, Stuerga Didier

**Affiliations:** 1 Department Interfaces, Laboratoire Interdisciplinaire Carnot de Bourgogne, UMR CNRS 6303, UBFC, Dijon, France; 2 Materials, Environments and Contamination Control Section, ESTEC – European Space Research and Technology Centre, Noordwijk, Netherlands

**Keywords:** Physical chemistry, Surface chemistry, Characterization and analytical techniques

## Abstract

Organic contamination is a major problem in the space industry, particularly for satellite optical devices. Although the standards governing this problem are extremely strict, there is no efficient method to measure the in situ, real-time deposition of organic contaminants. An original method based on thickness measurement is reported and tested in this paper. The use of microwave transduction allows to estimate the thickness of paraffin oil and silicone oil deposits from a few nanometers to several hundred nanometers in a low-pressure (mbar) environment. A linear response of the microwave sensor as a function of contaminant layer thickness is thus evidenced for both silicone and paraffin oils, paving the way for the integration of this method as an on-board contamination detection device.

## Introduction

In a fast-growing field such as space, controlling organic contamination of satellites and optical devices is mandatory, at every stage of their development and assembly, but also during flight. Indeed, contaminating layers can lead to equipment dysfunction or render measurements irrelevant. Standards for monitoring contamination are increasingly strict (ECSS-Q-ST-70-05 C, ESA Standard) aiming for tolerance levels in the nanometer range^1,2^. Current monitoring methods based on FTIR measurements allow the punctual and ex situ estimation of contamination levels. No dedicated methods allow to measure in situ and in real-time the contamination deposition^3^. The current technique lacks reliability and the ability to provide real-time characterization, leading to limitations in assessing the contamination of flight hardware during their lifetime.

Newly developed and future instruments display more and more sensitivity to molecular contamination. Typical detrimental effects are, among other, signal transmission loss, laser-induced contamination or damage on optics due to the absorption of the contamination layer depending on its chemical composition.

In this framework, this paper reports an ambitious project carried out in collaboration with the European Space Agency (ESA) concerning the use of microwave transduction to characterize a contaminant layer. This technique allows the development of a sensor, easily integrated, and miniaturized, able to characterize the thickness of contamination layers in situ and in real time. Consequently, this work represents a multidisciplinary challenge. The pollutants to be detected are hydrocarbons and silicones such as paraffin oil and silicone oil. At this stage, this paper constitutes a proof of concept, but does not comply with the strict standards governing the space industry. All this work has been designed to take a step forward regarding constraints ESA has to face. The hardware was selected on the basis of its on-board suitability and stability, while the protocols put in place included measurements in a low-pressure environment (a few mBar) with the aim of approximating flight conditions.

One key point is the determination of the amount of pollution, achievable through the evaluation of the thickness of contaminants in presence. To do so, a large number of thickness characterization techniques are available^4^. What differentiates these techniques from one another is mainly the different thickness ranges and the type of layers to be characterized (physical state, homogeneity, conductivity). Besides, these are “laboratory” techniques and are not suitable for on-board equipment. These laboratory thickness characterization techniques and their specificities are summarized in Table 1.

**Table 1:** Common thickness characterization techniques: practical advantages and limitations.

Techniques	Type of layer	Limits	Range
AFM^5,6^	Solid	Need a solid layer	Few nm to several µm
Optical microscopy^7^	Solid/liquid	Can’t observe objets smaller than the wavelenght of visible light	> 400 nm
	Inhomogeneous layer		
Ellipsometry & Reflectometry^7,8^	Solid/liquid	Homogeneous layer only. Need to know the refractive index of the layer	Few nm to hundreds of µm
X-Ray reflectivity^6,9,10^	Solid	Need to know the exact composition of the layer to process the data	Few nm to 1 μm
TEM^6^	Solid	Destructive method. Heavy sample preparation	0.1 nm to several µm
SEM^6^	Solid	Need a conductive, or semi-conductive solid layer, otherwise the layer needs to be metalized. Destructive method	nm to several µm
XPS^11,12^	Solid	Need a solid layer and very thin layer	< 10 nm

**Fig. 1:**
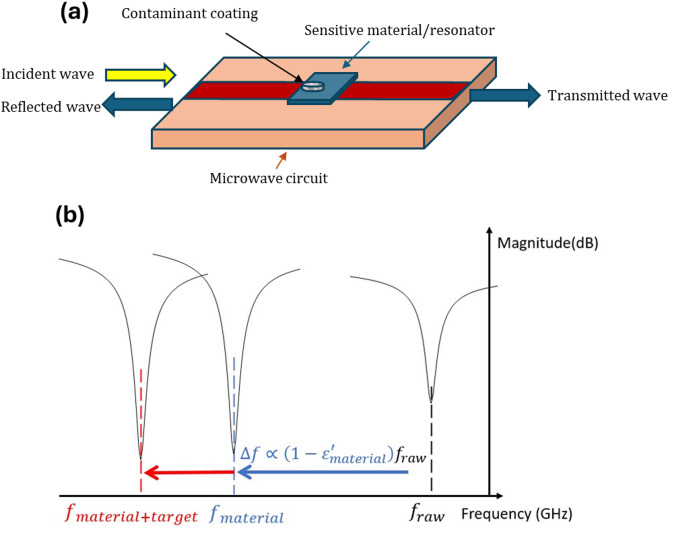
Microwave transduction principle (**a**) microwave transduction principle: measurement of microwave parameters (via incident/reflected waves) of a resonant circuit covered with a layer of sensitive material and the target to be detected. (**b**) Theory of small dielectric perturbations: the circuit has its own resonant frequency (f_raw_, in black), frequency shift towards low frequencies after deposition of the sensitive material (f_material_, in blue), new frequency shift towards low frequencies after absorption of the target (f_material+target_, in red)

Among all techniques available for detecting or characterizing physico-chemical processes, microwave transduction is relatively recent and has been particularly developed since the last 20 years^13–20^. Among other, it offers advantages highly complementary to conventional transduction techniques. The principle of microwave transduction is based on changes in the dielectric permittivity of a sensitive material deposited on the surface of a sensor^21^. In the presence of the target, variations in permittivity result in changes of the resonant frequency measured by a device interrogating the sensor. Figure 1 illustrates the principle of microwave transduction. One of the advantages of microwave transduction is its ability to provide quantitative results^22,23^. Indeed, the theory of small dielectric perturbations predicts that frequency variations are proportional to the variation in permittivity, and therefore to the quantity of target adsorbed on the sensor surface^24,25^. In addition to the quantitative aspect, electronics required for microwave transduction are compatible and adapted to all IoT and 5G-related issues^26,27^.

To develop the use of microwave transduction for contamination measurements, three major steps are mandatory and will be described in the Methods section, together with the used material. The first step is the generation of nanometric and inhomogeneous contaminant layers on the surface of microwave sensors. These layers have to present characteristics close to those of the undesirable deposits identified by ESA on space equipment. The second step concerns the thickness of the deposits which we must be able to estimate. Finally, the last step is the sensor response, through the measurement of the frequency shift induced by the presence of the deposit. Ideally, the characterization of the thickness of these layers by microwave transduction will be considered effective and relevant when a straight line is obtained linking the microwave variations (step 3) to the estimated thickness of the layer (step 2).

## Results

**Fig. 2:**
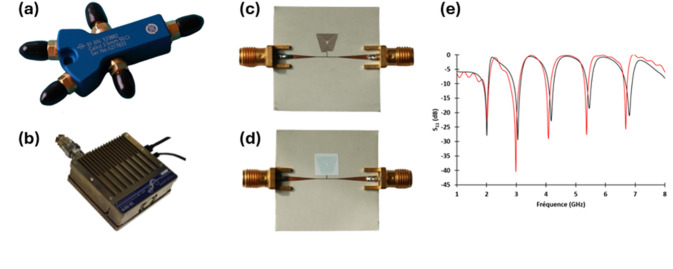
Embedded microwave equipment used and sensor response. (**a**) Calibration kit used KitCal 3.5 mm 50 Ω. (**b**) Reflectometer used to measure microwave parameter R180 CopperMountain. (**c**) Microwave resonator without sensitive material. (**d**) Microwave resonator with a patch of TiO_2_ (doctor blade deposition technique). (**e**) Sensor response after deposition of sensitive material obtained by simulation (red) and measurement (black)

In accordance with the methods described in the dedicated section, the first step is calibration. To do so, the sensor is covered with a layer of sensitive material, conditioned, weighed a first time and then the initial microwave response is measured. After which a layer of contaminant is deposited, and the new microwave response is measured. Finally, a second weighing is performed. Figure 2a,b presents the calibration kit and the reflectometer used in this work to measure the microwave parameters. Additionally, Fig. 2 also shows the raw sensor (c), the sensor covered with a sensitive material layer (d), and the corresponding sensor response (e).

**Fig. 3:**
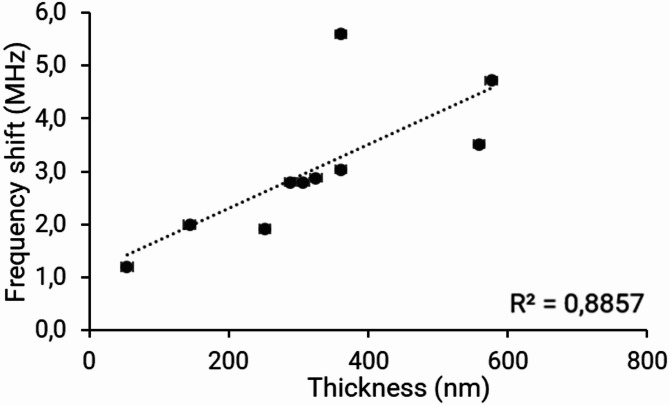
Frequency variations (in MHz) as a function of paraffin oil layer thickness (in nm).

**Fig. 4:**
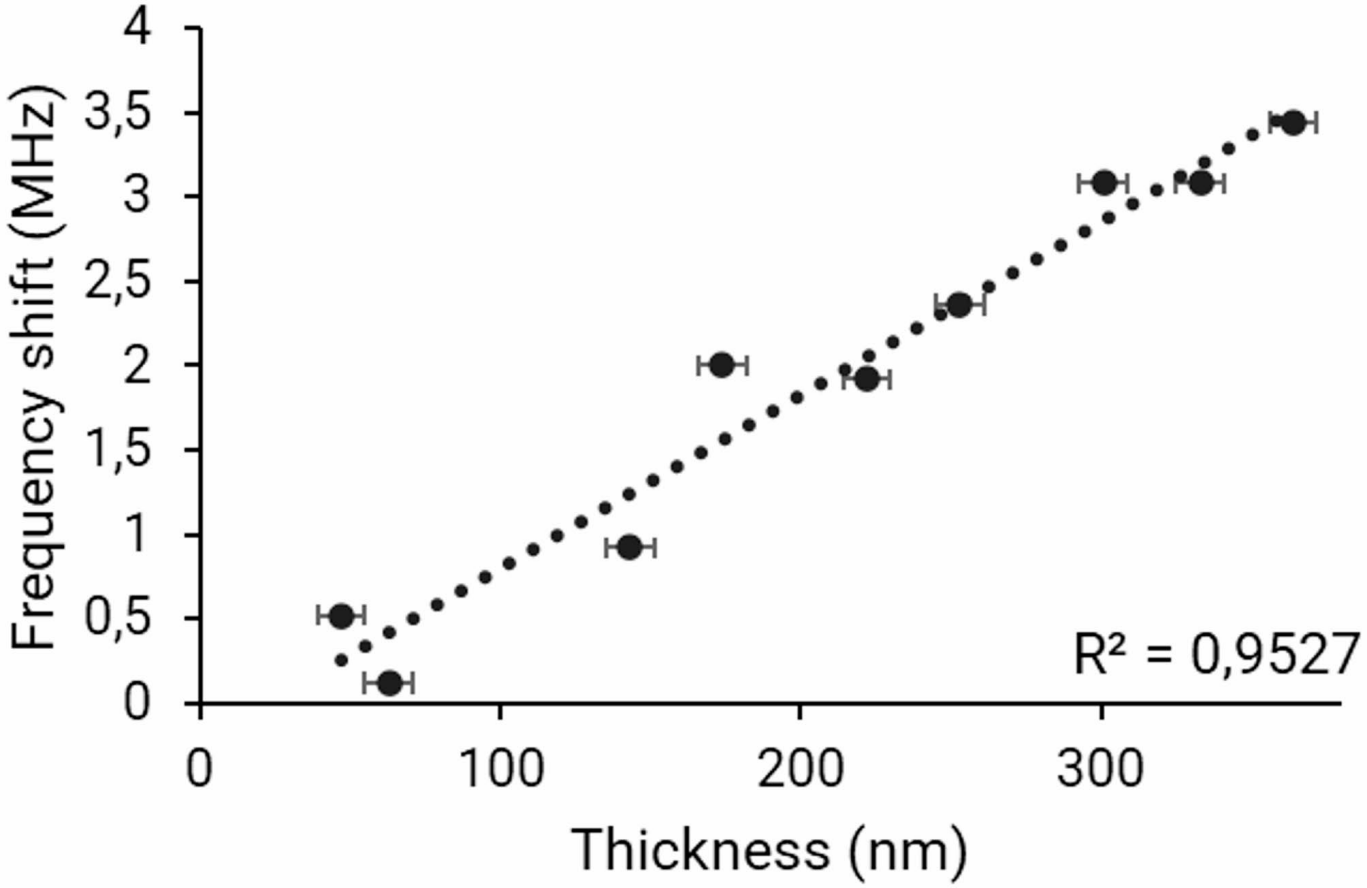
Frequency variations (in MHz) as a function of silicone oil layer thickness (in nm).

The results presented in Figs. 3 and 4 were obtained at the resonance frequency centered around 4.12 GHz. The evolution of frequency shifts as a function of estimated contaminant (paraffin oil) thickness is thus reported on Fig. 3, where each point is obtained from a single sensor.

According to these results, a linear evolution is observed between measured frequency variations and the thickness of the contaminant layer (R^2^ = 0.88). This demonstrates the quantitative aspect of microwave characterization of deposited layers. The deviation from linearity is largely explained by the experimental point at 380 nm thickness, which appears to be an outlier. It has been determined that this outlier originated from a defect in one of the sensor’s electronic connector solder joints.

Same experiments have been performed with silicone oil as contaminant. Related results are reported on Fig. 4.

The results for this contaminant also show linearity between measured microwave variations and contaminant layer thickness (R^2^ = 0.95), confirming the relevance of our method.

To obtain the estimated thicknesses of the contaminant layers, a calculation was performed taking into account the mass of contaminant deposited on the sensor, the density of the contaminant, and the surface area of the sensor. Tables 2 and 3 below provide details of this correspondence.

**Table 2:** Correspondence between paraffin oil mass deposited and estimated thickness.

Paraffin oil	Mass deposited (10^− 5^ g)	Estimated thickness (nm)
	14	289
	15	307
	12	252
	18	361
	7	144
	3	54
	28	559
	18	361
	28	577
	16	325

**Table 3:** Correspondence between silicone oil mass deposited and estimated thickness.

Silicone oil	Mass deposited (10^− 5^ g)	Estimated thickness (nm)
	3	47
	4	63
	14	222
	9	143
	21	333
	19	301
	23	364
	11	174
	16	253

## Methods

The description of the experimental part can be divided into several categories, taking into account both the equipment used to carry out this work (microwave sensor, hermetic cell, reflectometer) and the various protocols developed to obtain reproducible results. These protocols need to be robust, since in this work, each experimental point is obtained from a single sensor. In other words, to obtain the results presented here, several dozen different sensors had to be used. So, to avoid any loss of precision, each step, from sensor design and packaging, through deposition of the sensitive material on its surface, to characterization of the thickness of the contaminant layer, must be meticulously controlled.

### Microwave sensor: trapezoidal multiresonator

The microwave sensor used is a trapezoidal multi-resonator with resonant frequencies evenly distributed from 1 to 8 GHz^28^. The advantages of this resonator are manifold. Firstly, the device offers the possibility of broadband measurement to increase the probability of observing the phenomenon of interest, namely the frequency dependency. In addition, the theory of small dielectric perturbations predicts that the higher the resonant frequency, the greater the frequency variations due to adsorptions (or desorptions) at the sensor surface. As a result, the sensor can be used to select the highest resonance frequency where the phenomena are observed, in order to measure the greatest frequency variations. The final advantage of the multi-resonator is the possibility of multiple data for a single measurement. Depending on the analysis frequency range, the measured phenomenon can be observed at the “optimal” resonance frequency (presenting the greatest variations), but also at the sensor’s other resonance frequencies (particularly useful for AI data processing, dataset construction).

The sensors used in this work were produced by industrial processes in order to minimize the differences in raw response between each resonator and thus increase reproducibility.

### Sensitive material: TiO_2_ P25 patch

To maximize measured frequency variations, a layer (or patch) of P25 titanium dioxide is deposited on the surface of the microwave sensor^29^. Deposition is carried out by dropcasting (drop volume = 70 µL) on the resonant part only (spiral, using a deposition mask), while the rest of the sensor is protected. Following deposition, the sensors are conditioned for 18 h in an oven (60 °C) to induce maximum desorption of water (water due to deposition, but also atmospheric water). The great influence of variations in the amount of water on the sensor surface on microwave measurements has already been demonstrated in other works^23^.

### Environment: reflectometer and cell

Experiments are carried out in a hermetically sealed, circular stainless steel cell of 20 cm diameter. The hermeticity of the cell guarantees freedom from external interference such as ambient air, humidity or other gaseous interferents. Microwave measurements are carried out by a Copper Mountain R-180 reflectometer (1 port, 10 MHz − 18 GHz, 14.2 × 12.6 cm). The advantage of this instrument is that it offers good performance while being small enough to be portable. A calibration step is necessary before each measurement campaign^30^. This calibration was carried out under the same experimental conditions as the in situ sensor at *P* = 30mbar (see next section).

As the aim of this work is to determine the microwave modifications (dielectric permittivity, frequency shift) due to the presence of a contaminant layer, it is necessary to carry out a “before/after” measurement of the resonator’s microwave parameters. To achieve this, these measurements must be carried out directly in situ, taking care to maintain the same experimental conditions in both cases.

A first measurement (“before” measurement) of the sensor’s microwave parameters is taken at the end of the 18 h conditioning period after deposition of the sensitive material, while a second measurement (“after” measurement) is taken just after contamination of the sensor. In both cases, microwave measurements are taken at a pressure of *P* = 30 mbar.

### Sensor contamination protocol

Two different contaminants are studied in this work: a paraffin oil (medicated solution, COOPER) and a silicone oil (low viscosity silicone oil, 10cst, Sigma Aldrich). The technique chosen for the submicron deposits was evaporation-redeposition of the two oils. A crucible containing the contaminant is placed inside the closed cell housing the sensor, which is fixed in one position and whose surface is protected except for the resonant part (already covered with the sensitive material). A hot source is brought into contact with the crucible to raise its temperature locally, and a vacuum is drawn. This protocol induces evaporation of the contaminant. Once evaporated, the contaminant is redeposited inside the cell, particularly on the sensor coil (resonant part).

This protocol was optimized in the course of our work, and several parameters were studied: the positioning and orientation of the crucible in the cell with respect to the sensor, the pressure value, the temperature of the hot source and the initial quantity of contaminant to be introduced into the crucible. In particular, we determined that a minimum pressure was required to ensure minimum convection of the contaminant in the closed cell, otherwise the deposit would not be achieved. This pressure was estimated at 30 mbar. The variation in deposition time results in a variation in the thickness of the deposited contaminant layer.

For paraffin oil, the following experimental conditions were defined: *P* = 30 mbar, initial mass in the crucible m = 3.20 mg, hot-source temperature T = 350 °C, variable deposition time t = 15 s to 300 s.

For silicone oil, the following experimental conditions were defined: *P* = 30 mbar, initial mass in the crucible m = 3.00 mg, hot-source temperature T = 300 °C, variable deposition time t = 15 s to 300 s.

### Protocol for estimating the thickness of the deposited contaminant layer

Among the various thickness estimation techniques tested (ellipsometry, mechanical profilometer, atomic force microscopy), mass difference estimation was chosen. The principle is simple: a sensor mass measurement is taken before the contaminant layer is deposited (and after conditioning in the oven), then a second mass measurement is taken after deposition of the contaminant layer. Knowing the density of the deposited material and the surface area of the contaminant layer, it is possible to estimate its thickness.

This value corresponds to an “average” thickness over the entire surface, while optical microscopy images demonstrate that the contaminant layer is inhomogeneous on the surface of the sensor (alternating between islands of large drops of contaminant and uncovered surfaces).

The balance used must meet a number of criteria: it must be as accurate as possible, while still being able to measure an object the size of the sensor (2 × 3 cm). In our case, a KERN ABT 220-5DNM balance accurate to 0.01 mg was used. Mass measurements are taken after a vacuum of 30 mbar for 2 min.

## Conclusion

Organic contamination is a major problem in the space industry, particularly affecting the performance of instruments such as satellites. Despite strict standards, there is currently no method for in situ, real-time measurement of these deposits. Against this background, this work explored an innovative approach based on microwave transduction to characterize molecular contamination.

The results obtained demonstrate linearity between the thickness of the contaminating layers (paraffin and silicone oils) and the frequency variations measured. Although the thicknesses estimated in this work are higher than the characterization expectations laid down in the standards, the results validate the approach and the effectiveness of the method.

This proof-of-concept represents a significant advance towards reliable, in situ, real-time quantification of contaminant deposits in conditions representative of the space environment, using on-board equipment. Ultimately, this technology could improve the cleanliness control of sensitive devices and optimize their durability on mission.

## Data Availability

The datasets generated and/or analysed during the current study are not publicly available due to non-disclosure clause from ESA but are available from the corresponding author on reasonable request.
